# Oncological Outcomes of Metastasis-Directed Therapy in Oligorecurrent Prostate Cancer Patients Following Radical Prostatectomy

**DOI:** 10.3390/cancers12082271

**Published:** 2020-08-13

**Authors:** Gaëtan Devos, Charlien Berghen, Henri Van Eecke, Arthur Vander Stichele, Hendrik Van Poppel, Karolien Goffin, Cindy Mai, Liesbeth De Wever, Maarten Albersen, Wouter Everaerts, Gert De Meerleer, Steven Joniau

**Affiliations:** 1 Department of Urology, University Hospitals Leuven, Leuven 3000, Belgium; Henri.Vaneecke@student.kuleuven.be (H.V.E.); Arthur.vanderstichele@uzleuven.be (A.V.S.); Hendrik.vanpoppel@uzleuven.be (H.V.P.); maarten.albersen@uzleuven.be (M.A.); Wouter.everaerts@uzleuven.be (W.E.); 2 Department of Radiation Oncology, University Hospitals Leuven, Leuven 3000, Belgium; Charlien.berghen@uzleuven.be (C.B.); Gert.demeerleer@uzleuven.be (G.D.M.); 3 Department of Nuclear Medicine, University Hospitals Leuven, Leuven 3000, Belgium; karolien.goffin@uzleuven.be; 4 Department of Radiology, University Hospitals Leuven, Leuven 3000, Belgium; Liesbeth.dewever@uzleuven.be (L.D.W.); Cindy.Mai@uzleuven.be (C.M.)

**Keywords:** metastasis directed therapy, oligometastatic prostate cancer, oligorecurrent prostate cancer, metastasectomy, SBRT, salvage lymphadenectomy

## Abstract

Several retrospective and a few prospective studies have shown that metastasis-directed therapy (MDT) could delay clinical progression and postpone the initiation of systemic treatment in oligorecurrent prostate cancer (PCa) patients. However, these endpoints are strongly influenced by variables such as concomitant use of androgen deprivation therapy (ADT) and follow-up imaging protocols. The aim of this manuscript was to assess palliative ADT- and metastatic castration-resistant prostate cancer (mCRPC)-free survival as long-term oncological outcomes in oligorecurrent PCa treated by MDT. We retrospectively identified consecutive post-prostatectomy oligorecurrent PCa patients treated by MDT (salvage lymphadenectomy, radiotherapy, or metastasectomy) at our tertiary referral center. Patients were eligible for inclusion if they developed recurrence following radical prostatectomy, had ≤5 metastatic lesions on imaging and had a serum testosterone >50 ng/dL or a testosterone suppression therapy-free interval of >2 years prior to the first MDT as an assumption of recovered serum testosterone (if no testosterone measurement available). Patients with castration-resistant or synchronous oligometastatic PCa at the time of first MDT were excluded. Repeated MDTs were allowed, as well as a period of concomitant ADT. Kaplan–Meier analyses were performed to assess palliative ADT-free and mCRPC-free survival. We identified 191 eligible patients who underwent MDT. Median follow-up from first MDT until last follow-up or death was 45 months (IQR 27–70; mean 51 months). Estimated median palliative-ADT free survival was 66 months (95% CI 58–164) and estimated median mCRPC-free survival was not reached (mean 117 months, 95% CI 103–132). In total, 314 MDTs were performed and 25 patients (13%) received ≥3 MDTs. This study demonstrated that (repeated) MDT is feasible and holds promise in terms of palliative ADT-free and mCRPC-free survival for patients with oligorecurrent PCa. However, these findings should be confirmed in prospective randomized controlled trials.

## 1. Introduction

The optimal treatment strategy in oligometastatic prostate cancer (PCa) patients following primary treatment is not yet determined and remains challenging [1,2]. With the introduction of novel imaging techniques such as choline and PSMA PET/CT at the time of biochemical recurrence (BCR), more patients are now diagnosed with oligorecurrent PCa (defined as up to five lesions on imaging), leading to a shift in the treatment paradigm towards metastasis-directed therapy (MDT) [3]. However, the long natural history of PCa makes it difficult to determine the effect of novel treatment options on hard endpoints in clinical trials. According to the Intermediate Clinical Endpoints of Cancer of the Prostate (ICECaP) Working Group, metastasis-free survival is a good surrogate for overall survival in localized and non-metastatic castration-resistant PCa (CRPC) [4,5]. However, in oligorecurrent castration-sensitive PCa treated with MDT, the ideal intermediate endpoint has not yet been determined as long-term outcome data on cancer-specific and overall survival are lacking. Several retrospective and few prospective studies have shown that MDT could delay both clinical progression and the initiation of androgen deprivation therapy (ADT) in oligorecurrent PCa [6,7,8,9,10,11,12,13,14,15]. However, these endpoints are strongly influenced by variables such as concomitant use of ADT, follow-up imaging protocols and imaging techniques. For example, the randomized phase-2 ORIOLE trial, the single-arm prospective POPSTAR study, and the randomized phase-2 STOMP trial, demonstrated that MDT postponed progression in patients with oligorecurrent PCa (defined as up to 3 lesions) [6,14,15]. Clinical progression, however, was assessed by different imaging techniques in those studies (conventional imaging vs. choline PET/CT). The STOMP trial also demonstrated that MDT postpones the initiation of ADT in patients with oligorecurrent PCa when compared to the surveillance arm. Yet, currently ongoing prospective trials allow concomitant ADT during MDT (NCT03569241, NCT02274779, and NCT03940235), specifically when radiotherapy (RT) is used because of the radiosensitizing effect of ADT and/or the elimination of micrometastases not yet visible on imaging [16,17]. Therefore, a more clinically relevant endpoint might be the postponement of initiation of palliative, life-long ADT. Also, the onset of metastatic CRPC (mCRPC) appears to be an objective endpoint and has already been shown to be a good surrogate for overall survival in post-prostatectomy patients with second biochemical recurrence following salvage RT [18]. Moreover, these endpoints are also economically interesting as postponement of the onset of palliative ADT and/or mCRPC, might save a huge amount of money and improve the patient’s quality of life [19,20].

In this retrospective cohort study, we report on long-term palliative ADT-and mCRPC-free survival of oligorecurrent post-prostatectomy patients treated by MDT at our tertiary referral center. As secondary outcomes, BCR-free, clinical recurrence-free, cancer-specific, and overall survival are reported.

## 2. Results

We identified 191 consecutive oligorecurrent (defined as ≤5 lesions on imaging) castration-sensitive post-prostatectomy patients treated with MDT (salvage lymph node dissection (sLND), metastasectomy or (stereotactic body) radiation therapy ((SB)RT)) between 2007 and 2019 at our institution. Table 1 provides baseline characteristics at the time of the initial diagnosis of PCa and radical prostatectomy (RP). A major proportion of patients had negative prognostic factors for recurrence at final pathology: 19.3% had positive lymph nodes (LN), 41.9% had ISUP (International Society of Urological Pathology) grade group disease ≥4 and 61.7% had tumor stage ≥pT3a. As a consequence, 65.5% of the patients received adjuvant/salvage RT following RP. Median time from RP to (second) BCR (prostate-specific antigen (PSA) >0.2 ng/mL) was 28.5 months (IQR 9–69.9 months). Median time from (second) BCR following RP to positive imaging was eight months (IQR 2–30.7 months).

Table 2 provides an overview of the patient characteristics at the time of the first MDT. Median time from RP to first MDT was 58 months (IQR 24.7–104.5; mean 71.4 months). Median follow-up from the first MDT until last follow-up or death was 45 months (IQR 27–70; mean 51 months). At the time of first oligorecurrence, a total of 350 lesions were detected on imaging. Most patients had recurrence confined to a maximum of three or less lesions (89.6%).

Table 3 provides more detailed information on the patient characteristics for each type of MDT.

### 2.1. Palliative ADT-Free and mCRPC Free Survival

**Figure 1:**
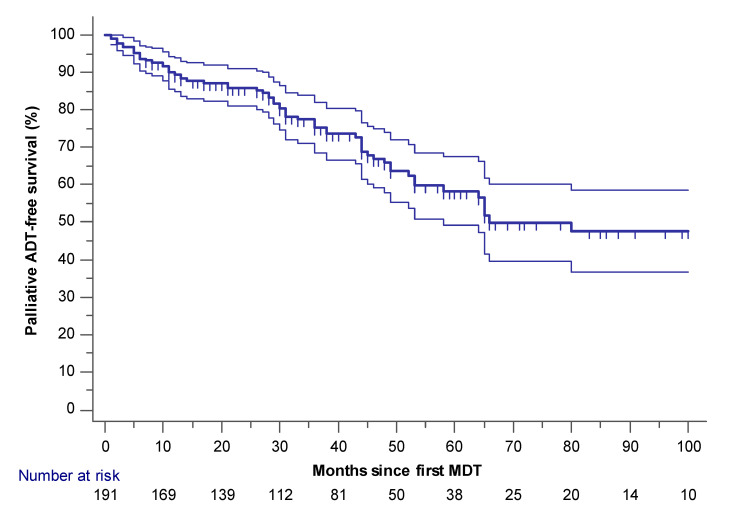
Palliative ADT-free survival (*n* = 191). Censored patients are marked with small vertical lines. The 95% confidence interval is provided (thin lines).

**Figure 2:**
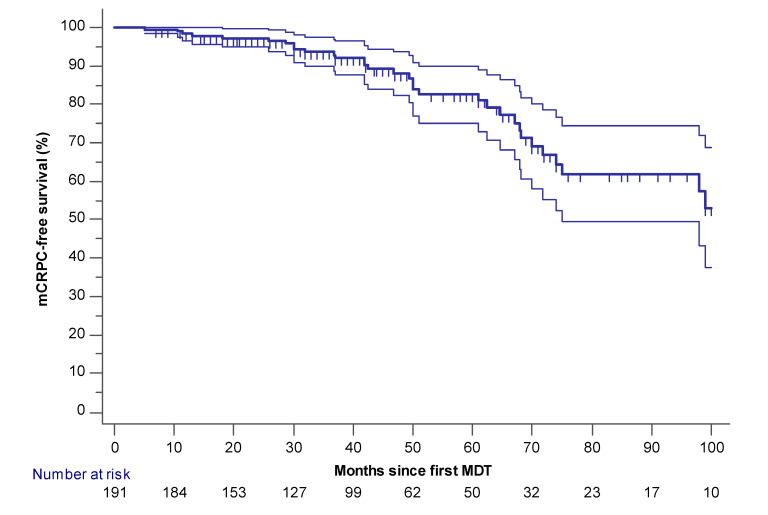
mCPRC-free survival (*n* = 191). Censored patients are marked with small vertical lines. The 95% confidence interval is provided (thin lines).

Estimated median palliative ADT-free survival was 66 months (95% CI 58–164)(Figure 1). Palliative ADT was initiated in 64 patients for the following reasons: >3 new or progressive lesions (*n* = 39), ≤3 new or progressive lesions but repeated MDT considered not feasible (*n* = 12) or rapidly rising PSA (PSA doubling time less than three months)(*n* = 13). Of the 64 patients initiated on palliative ADT, 32 developed mCRPC (median mCRPC-free survival not attained (mean 117 months 95% CI 103–132))(Figure 2). Median time from initiation of palliative ADT until the onset of mCRPC in these 32 patients was 15 months (IQR = 9–23.5; mean = 15.6). Estimated mCRPC-free survival at three- and five-year follow-ups were 93% and 82%, respectively. All mCRPC patients started next-line systemic treatment (enzalutamide, abiraterone, or docetaxel).

### 2.2. Multivariate Cox Proportional Hazard Regression Model Predicting Palliative ADT

At univariate analysis examining pre-MDT variables, PSA at the time of MDT, pN1 disease, pathological grade group ISUP (ISUP 5 vs. ≤4) [21], and type of lesion (M1a-c vs. N1) were significantly associated with palliative ADT following MDT (Table 4). At multivariate analysis, only pathological ISUP grade group 5 remained a significant predictor of palliative ADT (*p* = 0.002).

### 2.3. Secondary Endpoints

**Figure 3:**
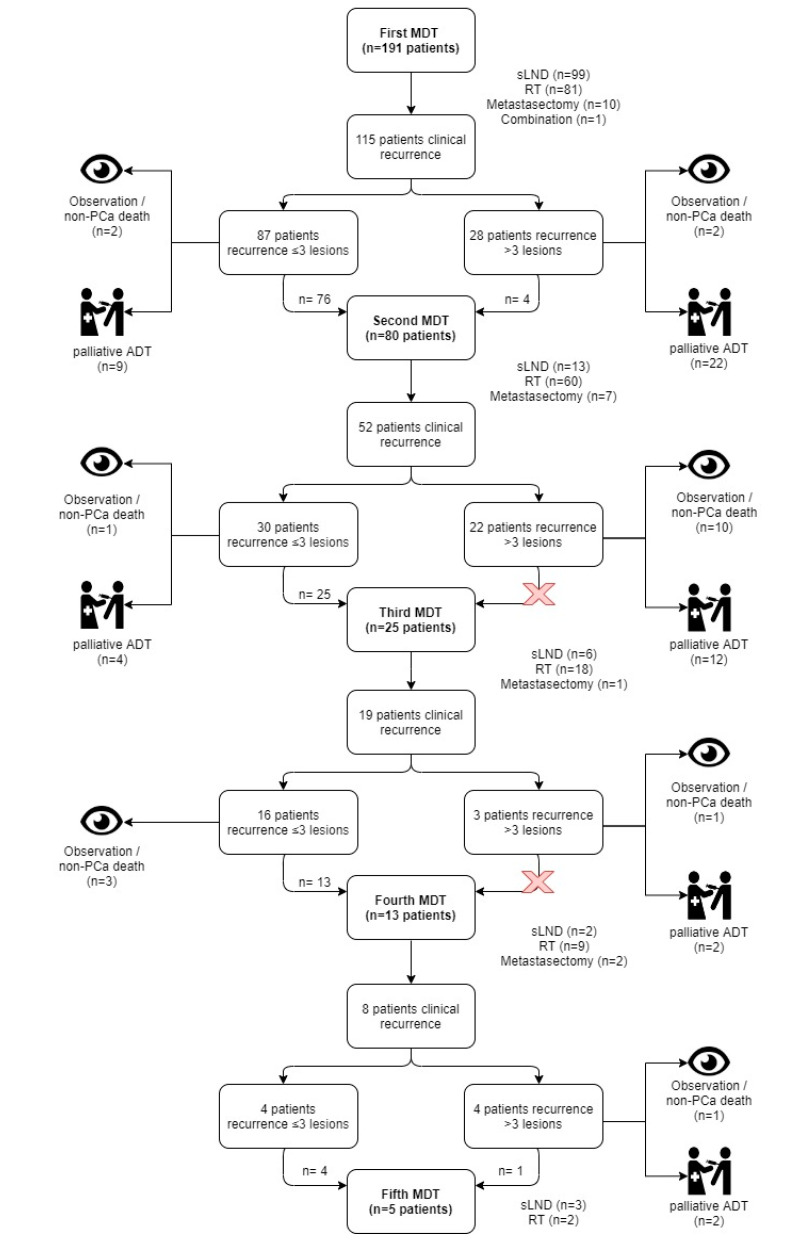
Breakdown of patients who received multiple MDTs. A distinction is made between patients with ≤3 new/progressive lesions and patients with >3 new/progressive lesions. ADT = androgen deprivation therapy; MDT = metastasis-directed therapy; PCa = prostate cancer; RT = radiotherapy; sLND = salvage lymphadenectomy.

Median BCR-free and clinical recurrence-free survival following MDT were eight months (95% CI 5–14) and 30 months (95% CI 25–38), respectively (Supplementary Figures S1 and S2). Of 115 patients who developed clinical recurrence following first MDT, PSMA and Choline PET/CT were used to detect disease recurrence in 79 (69%) and 28 (14.7%) patients, respectively. In total, 17 patients (8.9%) died, of whom 10 (5.2%) due to PCa (Supplementary Figures S3 and S4). Figure 3 provides a flowchart of clinical relapse patterns and type of repeated MDT. In total, 314 MDTs were performed and 25 patients (13.1%) received ≥3 MDTs. Forty-six patients (*n* = 46, 24.1%) who received an sLND or metastasectomy as first MDT, underwent repeated MDT at clinical recurrence. In total, 74 (38.7%) patients did not receive concomitant ADT during MDT (Supplementary Figure S5). In patients who received concomitant ADT (117 patients (61%), median time of concomitant ADT during MDT was 7 months (IQR 3–20). At the last follow-up, 30 patients (15.7%) had an undetectable PSA with a recovered testosterone (>50 ng/dL) level at a median follow-up of 46 months (IQR 32–71 months). Acute toxicity of first MDT can be found in Supplementary Table S1.

## 3. Discussion

In this manuscript, we described the long-term oncological outcomes, including palliative ADT-free and mCRPC-free survival, of oligorecurrent post-prostatectomy patients treated with MDT. At a median follow-up of 45 months (IQR: 27–70; mean 51 months (95% CI 47–56)), we observed an estimated median palliative ADT-free survival of 66 months. The median mCRPC-free survival was not reached. In our cohort, more than 65% of the patients received salvage (or adjuvant) RT following RP and consequently developed a “second” BCR before detection of oligometastatic PCa. The onset of metastatic CRPC may serve as a reliable surrogate for overall survival in post-prostatectomy patients with second BCR following salvage RT [18]. These findings on palliative ADT- and mCRPC-free survival are in strong contrast with the median time to BCR in our cohort which was only 8 months (and potentially less if no concomitant ADT would have been used at time of MDT). Therefore, clinicians should counsel their patients that the finality of MDT is not to achieve an undetectable PSA but rather to postpone the onset of palliative ADT and mCRPC.

Several other observations of our study are interesting for clinical practice. Firstly, more than 40% of the patients received multiple MDTs and in total 314 MDTs were performed. We consequently strived for repeated MDTs if feasible in well informed and motivated patients. It was already demonstrated by Decaestecker et al. that repeated SBRT is feasible in oligorecurrent PCa [22]. However, our series also demonstrated that repeated MDT is feasible (and safe) in patients who received sLND or metastasectomy as 46 (24.1%) of these patients received repeated MDT. Patients were often eligible for repeated MDT when ≤3 new/progressive lesions on follow-up imaging were detected (Figure 3). Secondly, the dogma of metastatic disease being incurable is challenged with our data as 15.7% (*n* = 30) had an undetectable PSA with a recovered testosterone (>50 ng/dL) level at last visit with a median follow-up of 46 months (IQR 32–71 months). Finally, we identified pathological ISUP grade group 5 as a negative predictor for palliative ADT at multivariate analysis. Similar to this, Fossati and colleagues observed that Gleason group 5 was associated with early clinical recurrence following sLND in oligorecurrent PCa [21]. Therefore, patients with ISUP grade group 5 at final pathology following RP may not be optimal candidates for MDT.

Long-term oncological outcomes of oligorecurrent PCa patients treated by MDT are scarce. One of the first studies looking at mCRPC-free survival was a multi-institutional retrospective analysis comparing SBRT with elective nodal RT [23]. With a median follow-up of 3 years, mCRPC-free survival was almost 90% in both groups which is similar to our findings (93% at 3 years follow-up). In addition, an update of the STOMP trial showed that the 5-year mCRPC free survival was 76% in the MDT group which is also similar to our results (82% at five years) [24]. Suardi et al. reported long-term outcomes of 59 oligorecurrent PCa patients treated by sLND with a median follow-up of more than 6 years [9]. Roughly 40% of these patients were clinical recurrence-free at long-term follow-up (8 years). This is superior to the clinical recurrence-free survival in our cohort (only 25% at 4 years follow-up)). This difference is explained by the fact that most patients (66%) in the Suardi study received long-term ADT, which contributed to the postponement of clinical recurrence. Bravi et al. reported long-term outcomes (median follow-up greater than 7 years) of 189 oligorecurrent PCa patients treated with sLND at 11 tertiary referral centers [25]. Median clinical recurrence-free survival was superior to our cohort (median 63 months vs. 30 months, respectively). Again, this difference is explained by the high proportion of patients receiving long-term (adjuvant/salvage). ADT within 6 months from sLND in their series (61%). Recently, the long-term outcomes of the phase-2, SABR-COMET trial were reported in which 99 oligorecurrent (≤5 lesions) patients, of whom 16 had oligorecurrent PCa, were randomized between standard of care or standard of care + SBRT [13]. At a median follow-up of 51 months, patients in the SBRT arm had improved overall survival (17% vs. 42.3%, respectively, *p* = 0.006). Interestingly, as in our cohort, many long-term survivors received repeated MDTs during follow-up.

There are some limitations to this study. Firstly, the patient characteristics of our cohort were heterogeneous in terms of MDT, type of imaging technique used prior to MDT, and imaging technique to assess recurrence following MDT. However, it was not the purpose of this study to compare different MDTs or imaging techniques. In addition, all patients received RP as primary treatment and most patients (>90%) were assessed with PET/CT prior to MDT and at the time of clinical recurrence following MDT. Secondly, we collected the number of months on concomitant ADT during MDT for each individual patient (Supplementary Figure S5). However, the time for testosterone to recover from these short-term treatments was likely longer than the total time on concomitant ADT. As a consequence, the effect of MDT itself in patients who received concomitant ADT was probably overestimated. Thirdly, this was a retrospective single-center study that is prone to several types of bias, especially selection bias. Furthermore, this study lacked an appropriate control group. Therefore, we are not able to compare oncological outcomes with patients treated with immediate systemic treatment or watchful waiting. Lastly, the observed median follow-up in our cohort was 45 months which is lower than the observed median palliative ADT-free (66 months) and mCRPC-free survival (median not attained). Therefore, outcomes should be interpreted with caution.

Notwithstanding these limitations, this retrospective cohort study represents, to our knowledge, the largest single-center series published with a median follow-up of 45 months (mean 51 months) and a total of 314 MDTs. In addition, for the first time in the literature, a detailed breakdown of repeated MDT, including sLND and metastasectomy, was provided. Lastly, this is one of the first series reporting on mCRPC-free and palliative-ADT free survival which are more reliable endpoints compared to BCR-free, clinical recurrence-free- and ADT-free survival in absence of long-term survival data.

## 4. Methods

We retrospectively collected data of all consecutive post-radical prostatectomy oligorecurrent PCa patients treated by MDT ((SB)RT, sLND, or metastasectomy) at our tertiary referral center between 2007 and 2019. Institutional ethical review board approval was obtained (S62433) prior to data collection. Patients were eligible if they developed clinical recurrence following RP (+/− adjuvant or salvage RT), had ≤5 metastatic lesions on imaging and a serum testosterone level >50 ng/dL at the time of inclusion. In case no testosterone level was available, patients were eligible for inclusion if testosterone suppression therapy-free interval was >2 years prior to the diagnosis of oligometastatic recurrence, hereby assuming recovered serum testosterone (>50 ng/dl). Castration-resistant and synchronous oligometastatic PCa patients at the time of the first MDT were excluded. The temporary use of concomitant ADT with (SB)RT was allowed, as well as repeated MDT in case of subsequent oligorecurrence. Decisions to perform MDT were uniformly made at the multidisciplinary board meetings and all patients were counseled regarding the investigational nature of the treatment.

### 4.1. Radiotherapy

Bone lesions were treated with SBRT. Lymph nodes metastases (N1, M1a disease) were treated with either a template elective field radiation (primary or post-sLND) or SBRT, depending on individual patient characteristics, patient’s preference, and technical possibilities.

#### 4.1.1. Stereotactic Body Radiation Therapy

SBRT was delivered to all visible metastatic lesions but with a maximum of three lesions at the same time. Computed tomography (CT) images (1 mm slices), together with T2-weighted magnetic resonance imaging and/or PSMA PET-CT images were used to optimize delineation. Gross tumor volume (GTV) was defined as all visible tumor based on the available imaging. There was no additional margin for microscopic tumor spread. In order to account for organ motion and setup error, margins of 3–7 mm were used to expand the gross visible tumor to define the planning target volume (PTV). The prescription dose was set at 30 Gy in three fractions of 10 Gy, separated by 48–96 h, prescribed to the periphery of the PTV at 80% of the maximal dose, with 100% of the dose covering 95% of the planning target volume. The dose constraints from the American Association of Physicists in Medicine Task Group 101 were used [26].

#### 4.1.2. Pelvic and/or Para-Aortic Lymph Node (PALN) Irradiation

Pelvic and/or PALN irradiation was applied to different scenarios:

1. Primary pelvic and/or PALN with a simultaneous integrated boost (SIB) on the visible LN.
2. Postoperative pelvic and/or PALN (adjuvant/salvage) after pelvic and/or peri-aortic LN dissection.

The elective pelvic LN template consisted of the obturator, internal and external iliac, presacral, and common iliac LNs. Details on delineation, and the definition of CTV and PTV can be found elsewhere [27]. If a positive LN was visible on PET-CT, this was considered as GTV and delineated separately. Details on delineation of the PALN can be found in our previous work [28]. A minimal dose of 45 Gy (25 fractions of 1.8 Gy) was prescribed as D99 (which is the dose received by 99% of the volume and acts as a surrogate for minimal dose) to the PTV of the pelvic LN and PALN. The GTV was treated to 62.5 Gy in those 25 fractions (SIB). The applied planning technology was IMAT/VMAT/RapidArc. Image-guided RT was performed using daily cone-beam CT [29].

### 4.2. Salvage Lymphadenectomy

The sLND template varied according to the location of the metastatic spots on preoperative imaging. The pelvic sLND template was defined as the removal of LN distal to the aortic bifurcation and retroperitoneal sLND template was defined as the removal LN above the aortic bifurcation till the level of the renal vasculature [30]. Templates were not limited to the positive spots on imaging and could be modified slightly according to the nodal recurrence site on pre-operative imaging, the extent of the prior pelvic LN dissection during RP, and to the degree of radiation fibrosis. For the robot-assisted procedures, the Xi Surgical System (Intuitive Surgical, Sunnyvale, CA, USA) was used with a six-port transperitoneal approach. The decision for adjuvant/salvage RT (+/− concomitant ADT) to the dissection template was made at the multidisciplinary oncology board meeting when pathology (positive section margins, extracapsular extension, and positive LNs) results were available.

### 4.3. Metastasectomy

These procedures were performed by experts of the affected organ (e.g., lobectomy by a thoracic surgeon, partial hepatectomy by a hepatobiliary surgeon) [31].

### 4.4. Follow-Up

Follow-up of patients included PSA testing and physical evaluation every 3–6 months. Testosterone was not routinely measured. Imaging was performed in case of rising PSA or at symptomatic progression. The best imaging technique available was used to detect recurrences.

### 4.5. Endpoints

The primary endpoints were palliative ADT- and mCRPC-free survival following MDT with mCRPC defined according to the European Association of Urology (EAU)-guidelines [32]. Secondary outcomes were BCR-free, clinical recurrence-free, cancer-specific and overall survival. Date of first MDT (day of surgery in case of sLND/metastasectomy or last day of radiation fraction in case of (SB)RT) was chosen as a starting point for follow-up and statistical analyses. Biochemical recurrence was defined as a PSA of 0.2 ng/mL or more following MDT. Clinical recurrence following first MDT was defined as the onset of new lesions or progression of treated lesions on imaging. Palliative ADT was considered as the initiation of life-long continued ADT. The number of months on concomitant (non-palliative) ADT was collected for each patient as well as reasons for starting palliative ADT. The toxicity of the first MDT was assessed as well.

### 4.6. Statistics

Kaplan–Meier analyses were used to determine BCR- and clinical recurrence-free survival rates (*n* = 139 and *n* = 115 events, respectively) and to assess palliative ADT-free and mCRPC free survival (*n* = 64 and *n* = 32 events, respectively). In order to identify patients who might benefit from MDT, pre-MDT variables were used for uni- and multivariate Cox proportional hazard regression to predict time to palliative ADT. Only significant variables (*p*-value < 0.05) at univariate analysis were used for multivariate analysis. Descriptive statistics were used for baseline patient characteristics (and toxicity assessment). Statistics were performed using the statistical software Medcalc, version 18.9 (MedCalc Software bvba, Ostend, Belgium; http://www.medcalc.org; 2018).

## 5. Conclusions

This study demonstrates that (repeated) MDT is feasible, safe, and promising in terms of palliative ADT-free and mCRPC-free survival for patients with oligorecurrent PCa. However, these findings should be confirmed in prospective randomized controlled trials.

## Figures and Tables

**Table 1:** Patient characteristics at the time of radical prostatectomy (*n* = 191).

**Median Age at Primary Treatment in Years (IQR; Mean)**	**61.3 (56–66; 60.9)**
**Median PSA at time of RP (IQR, ng/mL)**	9.2 (6.4–14.7)
<20 ng/mL	158 (82.7%)
≥20 ng/mL	25 (13.1%)
NA	8 (4.2%)
**Technique**	
Open	132 (69.1%)
Laparoscopy	13 (24.1%)
Robot	46 (6.8%)
**Concomitant pelvic LN dissection during RP**	
Yes	128 (67%)
No	63 (33%)
**Median number of LN removed during pelvic LN dissection (IQR)**	11 (6–20)
**Pathological N-stage**	
pN1	37 (19.3%)
**Pathological ISUP (%)**	
1	8 (4.2%)
2	42 (22%)
3	54 (28.3%)
4	43 (22.5%)
5	37 (19.4%)
NA	7 (3.6%)
**Pathological T-stage**	
pT2	68 (35.6%)
pT3a	57 (29.8%)
pT3b	59 (30.9%)
pT4	2 (1%)
NA	5 (2.6%)
**Positive section margins**	
R0	118 (61.8%)
R1	65 (34%)
NA	8 (4.2%)
**Adjuvant/salvage RT following RP**	
No adjuvant/salvage RT	66 (34.6%)
Adjuvant RT	15 (7.9%)
Salvage RT	110 (57.6%)
**Template adjuvant/salvage RT**	
Prostate bed	90 (47.1%)
Prostate bed + pelvic LN region	35 (18.3%)
**Concomitant ADT during adjuvant/salvage RT**	24 (12.6%)

Data are given as *n* (%) unless otherwise specified. PSA = prostate-specific antigen; ADT = androgen deprivation therapy; RP = radical prostatectomy; IQR = interquartile range; LN = lymph nodes; NA = not available, RT = radiotherapy, ISUP = international society of urological pathology.

**Table 2:** Patient characteristics at time of first MDT (*n* = 191).

**Median Age at MDT in Years (IQR; Mean)**	**66.9 (63–71.8; 67)**
**Median time from positive imaging to MDT (months, IQR)**	2.6 (1.7–4.7)
**Number of lesions on imaging (%)**	
1–3	171 (89.6%)
4–5	20 (10.4%)
**Type of imaging used (%)**	
^11^C-choline PET/CT	60 (31.4%)
^68^Ga- or ^18^F-PSMA PET/CT	113 (59.2%)
Bone scan and/or CT-scan	18 (9.4%)
**Type of recurrence (patient-based):**	
N1 *	101 (52.9%)
M1a	34 (17.8%)
M1b	45 (23.6%)
M1c	11 (5.8%)
**Location of lesions on imaging (lesion-based analysis)**	*n* = 350
Lymph nodes	**270**
Internal iliac	29
Obturator	35
External iliac	65
Common iliac	49
Presacral	24
Perirectal	11
Retroperitoneal	46
inguinal	7
Mediastinal	4
Supraclavicular	0
Skeletal	**69**
Appendicular	5
Axial	64
Visceral	**11**
**Any ADT prior to first MDT (e.g., during adjuvant/salvage RT)(%)**	
Yes	60 (31.4%)
Bicalutamide	22
LHRH agonist/antagonist	35
NA	3
No	131 (68.6%)
**Type of first MDT (%)**	
Radiotherapy-based	81 (42.4%)
Elective field radiation	31
SBRT	50
Metastasectomy	10 (5.2%)
sLND	99 (51.8%)
Adjuvant RT	11
Salvage RT	21
Combination: SBRT + sLND	1 (0.5%)
**Median PSA at time of first MDT (IQR, ng/mL)**	1.4 (0.6–3.4)

Data are given as *n* (%) unless otherwise specified. MDT = metastasis-directed therapy; RT = radiotherapy; SBRT = stereotactic body radiation therapy; sLND = salvage lymphadenectomy; PSA = prostate-specific antigen; IQR = interquartile range; ADT = androgen deprivation therapy; NA = not available. * N1-disease was considered as recurrence confined to the pelvic lymph nodes (obturator, internal iliac, external iliac, presacral, perirectal, and common iliac LN).

**Table 3:** Detailed information on first MDT.

**Salvage Lymphadenectomy (*n* = 100)**	
**Median PSA at time of sLND (IQR, ng/mL)**	1.4 (0.6–3)
**ASA classification at time of sLND**	
1–2	69 (69%)
3–4	16 (16%)
NA	15 (15%)
**Median BMI preoperative (IQR)**	26 (24–30)
**Median operation time (min, IQR)**	150 (120–190)
**Median blood loss (mL, IQR)**	200 (100–450)
**Hospital stay (IQR, days)**	5 (2–7)
Technique	
Open	65 (65%)
Robot	33 (33%)
Laparoscopy	2 (2%)
**Template**	
Pelvic sLND	63 (63%)
Retroperitoneal sLND	8 (8%)
Pelvic + retroperitoneal sLND	29 (29%)
**Median number of LN removed (IQR)**	19 (11–28)
**Median of positive LN at final pathology (IQR)**	2 (1–5)
**Adjuvant/salvage RT following sLND**	
Yes	32 (32%)
Concomitant ADT	31
Template pelvis	17
Template Pelvis + retroperitoneal LN	15
No	68 (68%)
**Radiotherapy (*n* = 82)**	
**Median PSA at time of RT (IQR, ng/mL)**	1.3 (0.45–3.95)
**Type of RT**	
SBRT	51 (62.2%)
Concomitant ADT	41
Median months concomitant ADT (IQR)	1 (1–1)
Elective field radiation	31 (37.8%)
Concomitant ADT	24
Median months concomitant ADT (IQR)	18 (6–24)
**Metastasectomy (*n* = 10)**	
**Median PSA at time of metastasectomy (IQR, ng/mL)**	3.4 (1.3–4.9)
**ASA-classification at time of metastasectomy**	
1	2 (20%)
2	7 (70%)
NA	1 (10%)
**Median BMI preoperative**	28 (25–29)
**Type of metastasectomy**	
Lung	6
Rectum	1
Testis	1
Liver	1
Adrenal gland	1
**Median hospital stay (IQR, days)**	4 (2–7)

Data are given as *n* (%) unless otherwise specified. ASA = American Society of Anesthesiologists ADT = androgen deprivation therapy; BMI = body mass index; MDT = metastasis-directed therapy; RT = radiotherapy; SBRT = stereotactic body radiation therapy; sLND = salvage lymphadenectomy; PSA = prostate-specific antigen; IQR = interquartile range; LN = lymph nodes. Note that the total number of first MDTs (*n* = 192) is larger than the number of patients (*n* = 191) as one patient received a combination of sLND and SBRT as the first MDT.

**Table 4:** Uni- and multivariate Cox proportional hazard regression models predicting palliative ADT following MDT in oligorecurrent PCa patients.

Variable	Univariate HR (95% CI); *p*-Value	Multivariate HR (95% CI); *p*-Value
**Number of lesions**		
4–5 lesions vs. 1–3 lesions	1.89 (0,89–4); 0.09	/
**Type of recurrence**		
M1a-b-c vs. N1	1.81 (1.09–2.98); **0.019**	1.88 (0.94–3.8); 0.07
**PSA at time of sLND**	1.08 (1.02–1.14); **0.006**	1.04 (0.97–1.12); 0.21
**pT-stage**		
pT3b-T4 vs. T2-T3a	1.5 (0.9–2.5); 0.11	/
**pN1 at RP**	2.26 (1.2–4.3); **0.01**	1.44 (0.7–2.9); 0.31
**Pathological ISUP grade group**		
ISUP 5 vs. ISUP ≤4	2.26 (1.32–3.8); **0.0028**	2.6 (1.4–5.1); **0.0029**
**Imaging technique prior to MDT**		
**No PSMA PET/CT vs. PSMA PET/CT**	1.32 (1.76–2.3); 0.31	/
**Time from primary treatment until first MDT**	1 (0.99–1.01); 0.2	/

MDT = metastasis-directed therapy; ADT = androgen deprivation therapy; CI = confidence interval; HR = hazard ratio; PCa = prostate cancer; RP = radical prostatectomy; PSA = prostate specific antigen; ISUP = International Society of Urological Pathology; sLND = salvage lymphadenectomy.

## Supplementary Materials

The following are available online at https://www.mdpi.com/2072-6694/12/8/2271/s1. Figure S1: Biochemical recurrence-free survival, Figure S2: Clinical recurrence-free survival, Figure S3: Cancer-specific survival, Figure S4 overall survival, Figure S5: Concomitant use of ADT during MDT. Table S1: Acute toxicity of first MDT.

## Abbreviations

ADT	Androgen Deprivation Therapy
BCR	Biochemical Recurrence
CI	Confidence Interval
CRPC	Castration-Resistant Prostate Cancer
CT	Computed Tomography
EAU	European Association of Urology
GTV	Gross Tumor Volume
HR	Hazard Ratio
ICECAP	Intermediate Clinical Endpoints of Cancer of the Prostate
IQR	Interquartile Range
ISUP	International Society of Urological Pathology
LN	Lymph Node
mCRPC	Metastatic Castration-Resistant Prostate Cancer
MDT	Metastasis-Directed Therapy
NA	Not Available
PCa	Prostate Cancer
PALN	Para-Aortic Lymph Node
PSA	Prostate-Specific Antigen
PSMA	Prostate Specific Membrane Antigen
PTV	Planning Target Volume
RP	Radical Prostatectomy
RT	Radiotherapy
SBRT	Stereotactic Body Radiation Therapy
SIB	Simultaneous Integrated Boost
sLND	Salvage Lymph Node Dissection/Salvage Lymphadenectomy
