# Supplementary material for: Oncological Outcomes of Metastasis-Directed Therapy in Oligorecurrent Prostate Cancer Patients Following Radical Prostatectomy
Source: Cancers (Basel). 2020 Aug 13;12(8):2271. doi: 10.3390/cancers12082271 (PMC7464259; doi:10.3390/cancers12082271)
Supplement: Supplementary file 1 [file cancers-12-02271-s001.docx]

| Supplementary table 1: Acute toxicity of first MDT | |
| --- | --- |
| Salvage lymphadenectomy (n=100) (Clavien-Dindo) | |
| Intraoperative  Venous injury  Ureteral injury  Rectum perforation  Chyle leakage  Nerve injury  Bladder perforation  Arterial injury  Postoperative complications (<90 days) following surgery:  Grade III-IV  Punction of lymphocele  DJ stent for hydronephrosis  Acute kidney injury (biopsy)  Active arterial bleeding: re-intervention  Urosepsis with intensive care stay  Splenectomy for low trombocytes.  Grade I-II  Lymphedema legs  Symptomatic lymphocele  Symptomatic scrotal edema  Wound infection/ flegmone  Ileus  Bladder infection  Symptomatic hematoma  Diarrhea  Trombocytopenia  Pneumonia  Veneus bleeding  Pyelonephritis  Urinairy retention  Umbilical hernia  Nausea  Hydronephrosis | 18 (18%)  9  2  2  2  1  1  1  41 (44%)  10 (10%)  3  3  1  1  1  1  31 (31%)  7  5  4  3  2  2  1  1  1  1  1  1  1  1  1  1 |
| Metastasectomy (n=10) (Clavien-Dindo) | |
| Intraoperative  Lungparenchym tear  Postoperative  Grade I-II  Pneumoniae  Chyle leakage | 1  2  1  1 |
| Radiotherapy (n=82) (CTCAE) | |
| Fractionated RT (n=32)  Grade I-II  Diarrhea  Urinary frequency  Fatigue  Proctitis  Sore throat  Urinary urgency  Paresthesia legs  Urinary incontinence  Nausea  Grade ≥III  SBRT (n=50)  Grade I-II  Urinary frequency  Diarrhea  Fatigue  Buttock pain  Hematuria  Grade ≥III | 15 (46,8%)  4  3  2  1  1  1  1  1  1  0  11 (22%)  4  2  2  1  1  0 |
| Data are given as n (%) unless otherwise noted. MDT= metastasis directed therapy; RT= radiotherapy; SBRT= Stereotactic Body Radiation Therapy; RT= Radiation therapy; | |

**Supplementary figure 1:** Biochemical recurrence-free survival. Censored patients are marked with small vertical lines. The 95% confidence interval is provided.

**Supplementary figure 2:** clinical recurrence-free survival. Censored patients are marked with small vertical lines. The 95% confidence interval is provided.

**Supplementary figure 3:** Cancer-specific survival. Censored patients are marked with small vertical lines. The 95% confidence interval is provided.

**Supplementary figure 4:** Overall survival. Censored patients are marked with small vertical lines. The 95% confidence interval is provided.

**
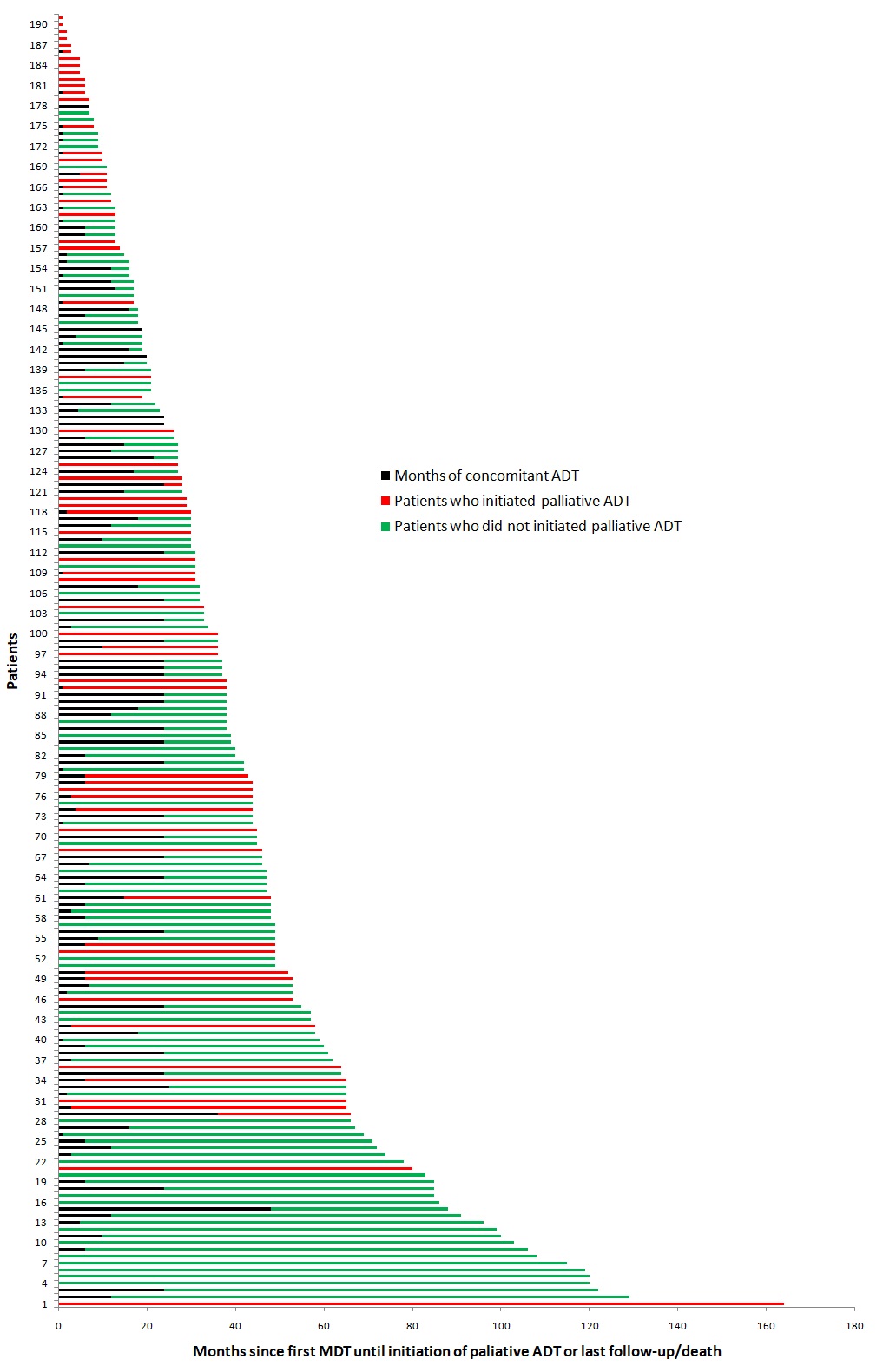
**

**Supplementary figure 5:** Each single bar represents a patient and the length of the bar represents the time in months since the first MDT until initiation of palliative ADT or last follow-up/death. A distinction is made between patients who initiated palliative ADT (red bars, n=64)and patients who did not yet started palliative ADT (green bars, n=127). The length of the black bar represents the total time on concomitant ADT during MDT. In total, 117 patients received concomitant ADT during MDT treatment.
